# Variation in colour markings of an unusual new *Asprothrips* species from China (Thysanoptera, Thripidae)

**DOI:** 10.3897/zookeys.716.20952

**Published:** 2017-11-23

**Authors:** Zhaohong Wang, Xiaoli Tong

**Affiliations:** 1 Department of Entomology, College of Agriculture, South China Agricultural University, Guangzhou 510642, China

**Keywords:** *Asprothrips*, Dendrothripinae, new species, thrips, Thripidae

## Abstract

The second species of the genus *Asprothrips* with a bicoloured body, *A. atermaculosus* **sp. n.**, is described and illustrated from China. This is characterised by considerable intra-population variation in the number and size of brown markings on the abdominal tergites. *Asprothrips fuscipennis* Kudô, previously described from Japan, is newly recorded in China.

## Introduction

**Figure 1.**
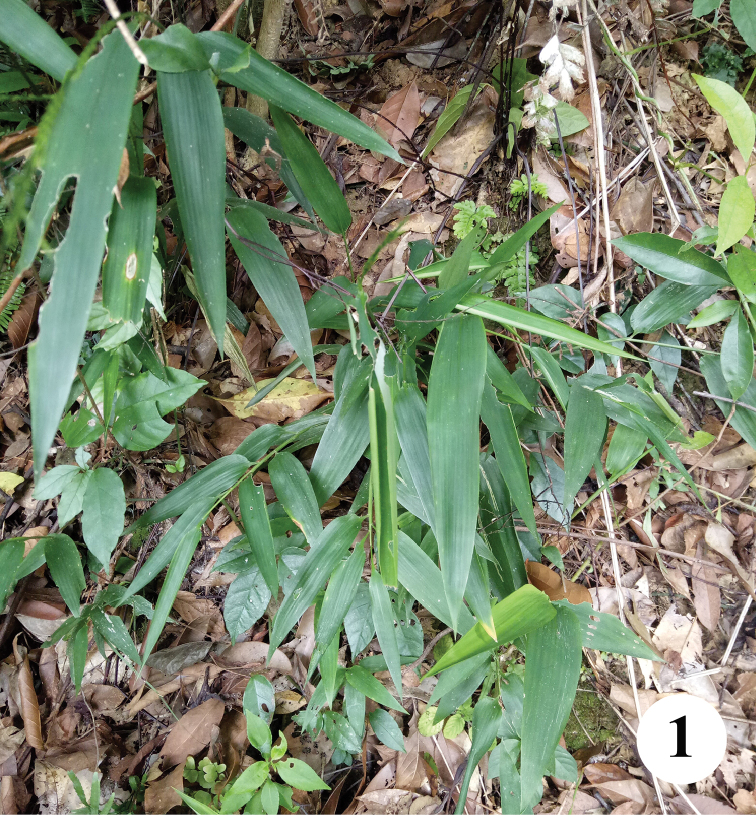
*Lophatherum gracile*, host plant of *Asprothrips atermaculosus* sp. n.

The genus *Asprothrips* Crawford, a small genus of the subfamily Dendrothripinae, currently comprises seven described species (ThripsWiki 2017), and Tong et al. (2016) briefly summarised the generic diagnosis and provided a key to the world species. *Asprothrips* species generally exhibit two colour types, with the body either brown or white (Mound 1999; Tyagi 2011). In contrast, Michel and Ryckewaert (2014a, b) described *A. bimaculatus* as the first bicoloured species in the genus. Here a second bicoloured *Asprothrips* species is described, which was collected from *Lophatherum gracile* (Fig. 1), a ubiquitous grass found throughout southern China. This thrips was collected during recent surveys on the thrips fauna of China, and *A. fuscipennis* Kudô that was previously known only from Japan is also recorded in China for the first time.

## Materials and methods

The thrips were collected by beating vegetation over a white plastic tray using a stick, and then sorted and preserved in 90 % alcohol. Examined specimens were mounted in Canada balsam using the method outlined by Zhang et al. (2006). Details of the morphological structures were examined with a ZEISS Imager A1 microscope, and the photos were taken by a Photometrics CoolSNAP camera. All type specimens are deposited in the Insect Collection, South China Agricultural University (**SCAU**).

## Taxonomy

### Asprothrips atermaculosus sp. n.

Taxon classificationAnimaliaORDOFAMILIA

http://zoobank.org/AEA30996-615F-4E7E-A000-21E3EC4AA61F

Figs 2–5 , 6–12 , 13–16

#### Material examined.

**Holotype** female (in SCAU): **CHINA**, Hunan province, Chaling County, Yunyangshan National Forest Park (26°47'58"N, 113°30'18"E, alt. 300m), collected from leaves of *Lophatherum gracile* (Poaceae), 8.viii.2017, leg. Zhaohong Wang.

**Paratypes** (in SCAU): 20 females, 25 males, taken with holotype. Fujian province, Sanming City, Sanyuan National Forest Park (26°10’N, 117°28’E, alt. 200m), 21 females and 31 males from leaves of *Lophatherum gracile* (Poaceae), 24.viii.2017, leg. Zhaohong Wang. Hunan province, Hengyang City, Mt. Hengshan (26°16'22"N, 112°42'22"E, alt. 530m), 2 females, collected from *L. gracile* (Poaceae), 6.viii.2017, leg. Zhaohong Wang.

#### Diagnosis.

Female body bicoloured, head, pronotum and antennal segments I–II brown; abdomen white except for the intra-population variation in the number and size of brown markings on the abdominal tergites I–VIII; fore wing white with two dark brown bands and the surface uniformly covered with microtrichia. Male body is similar to female in structure and colour pattern, but antennae white or yellowish white and the paired brown markings exist only in abdominal tergites I–II and VI; abdominal sternites III–VIII each with a small and oval pore plates.

#### Description.

**Figures 2–5.**
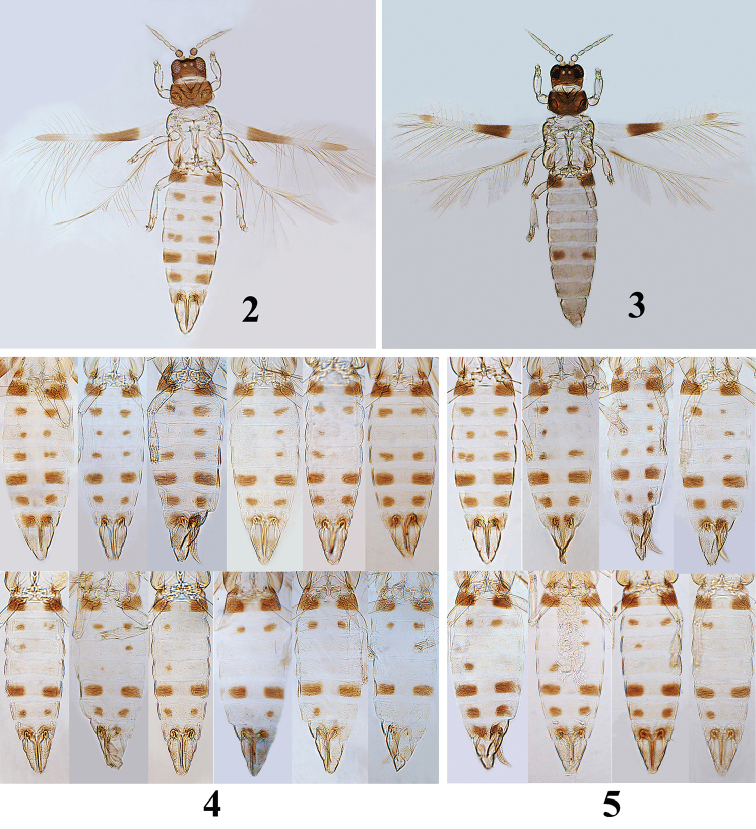
*Asprothrips atermaculosus* sp. n. **2** female **3** male **4** variation of colour markings on abdominal tergites in “Sanming” population **5** variation of colour markings on abdominal tergites in “Chaling” population.

Female (macropterous) (Fig. 2). Body bicoloured, dark brown and white; head and pronotum dark brown; antennal segment I pale brown, II dark brown, III–VIII white (Fig. 7); pterothorax and all legs white; fore wing white with two dark brown bands submedially and apically (Fig. 9); abdomen white except for tergites I–II and VI stably with the paired dark brown markings laterally, but those on tergites III–V and VII–VIII are variable individually in numbers and size within the same population as showed as figures 3–4, tergite IX white with a pale brown tint and X white.

**Figures 6–12.**
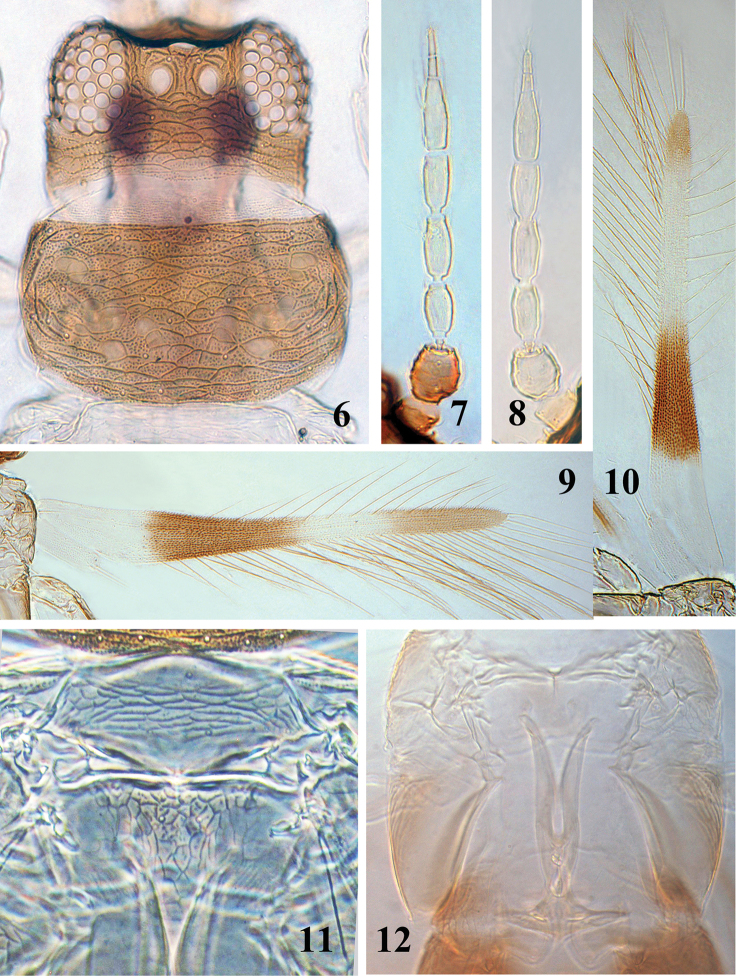
*Asprothrips atermaculosus* sp. n. **6** head & pronotum **7** antenna of female **8** antenna of male **9** fore wing of female **10** fore wing of male **11** meso- and metanotum **12** lyre-shaped metathoracic endofurca, female

*Head* (Fig. 6) approximately 2.0 times as wide as long; two pairs of minute ocellar setae present, pair II situated at middle between anterior ocellus and compound eye, pair III arising near anterior margin of posterior ocelli within ocellar triangle; four pairs of minute postocular setae present, first pair below the hind ocelli, second and third one near the compound eyes and fourth pair near the cheeks; vertex between eyes including ocellar triangle irregularly reticulate, occipital region of vertex reticulate with transverse dotted lines and internal granules within the reticules; cheeks serrated. Mouth cone short and rounded; maxillary palps three-segmented. Antennae 8-segmented (Fig. 7), antennal segment II large and globular with ridges on striae, III with a pedicel, III and IV each with a forked sense cone, V with a short simple outer sense cone, VI with three sense cones, inner one longest arising medially, reaching apex of segment VIII; microtrichia rows present on segments III–VI, III–V with three rows, VI with sparse microtrichia.

*Pronotum* (Fig. 6) approximately 2.0 times as wide as long, irregularly reticulate with numerous internal granules within the reticules; dorsal surface covered with approximately 26–32 short discal setae and four pairs of posteromarginal setae; ferna complete and narrower at middle. Mesonotum (Fig. 11) with transverse anastomosing striae without internal wrinkles or granules within the reticules, a pair of campaniform sensilla on anterior fourth, median setal pair situated submedially. Metanotum (Fig. 11) reticulate medially without granules within the reticules, median setae far back from anterior margin, campaniform sensilla present or absent. Metafurca bearing two lyre-shaped anterior arms extending into the mesothorax (Fig. 12). Fore wing uniformly covered with microtrichia (Fig. 9); fore wing apex with two long terminal setae, costa with 14–15 setae, first vein with 5–6 proximal and two distal setae, second vein with 4–5 setae; main posterior fringe hairs weakly wavy. Legs reticulate weakly; fore and mid tarsi 2-segmented, hind tarsus one-segmented; Hind tibiae with two apical stout setae.

**Figures 13–16.**
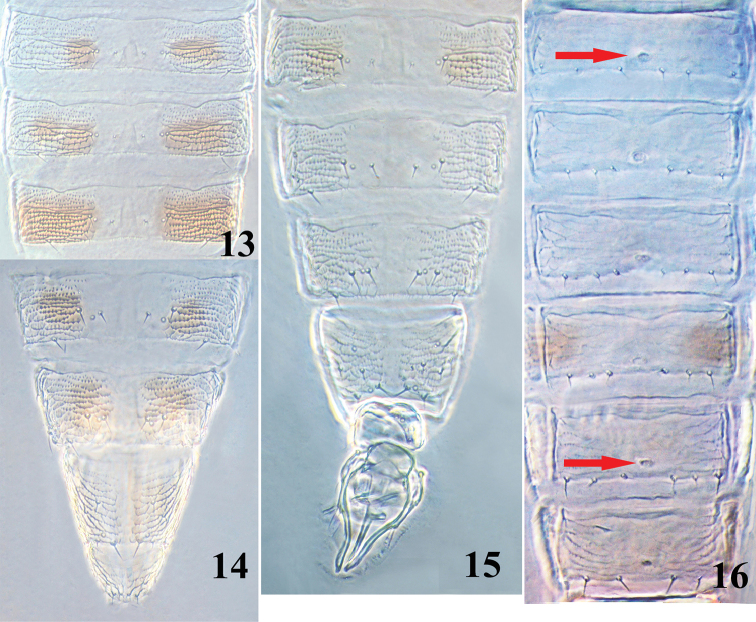
*Asprothrips atermaculosus* sp. n. **13** abdominal tergites IV–VI, female **14** abdominal tergites VII–X, female **15** abdominal tergites VI–X, male **16** abdominal sternites III–VIII, male

*Abdominal* tergites I–VII smooth medially between setal pair S2, with transverse sculpture lines bearing microtrichia laterally; S1 setae (median pair) on abdominal tergites II to VII small, the distance between their basal pores much greater than their length; paired campaniform sensilla between setae S1 and S2, much closer to S2 on tergites II–VII (Fig. 13); VIII–IX entirely covered with transverse sculpture bearing microtrichia except for groove medially; VIII with posterior marginal comb of small microtrichia only at middle; posterior margin of IX medially with a pair of fine and pointed setae directed medially (Fig. 14); tergite X without longitudinal dorsal split. Abdominal sternites II–VII weakly reticulate; II with one pair of setae and III–VII each with three pairs on posterior margin.

*Measurements* (holotype female in microns). Total distended body length 960. Head length (width) 63 (122); eye length (width) 47 (32). Pronotum length (width) 79 (147). Length of antenna 182; length (width) of antennal segments I 15 (20), II 22 (24), III 30 (14), IV 30 (13), V 26 (13), VI 36 (12), VII 10 (4) and VIII 13 (3). Fore wing length 1250.

**Male** (macropterous) (Fig. 3). Similar to female in structure and colour except for following characters: antennal segments I–II yellowish white (Fig. 8); fore wing with two brown bands submedially and apically, but the apical one much shorter than that of the female (Fig. 10); the paired brown markings exist only in abdominal tergites I–II and VI and without any markings on other tergites (Figs 3, 15); abdominal sternites III–VIII each with a small and oval pore plates (Fig. 16).

*Measurements* (paratype male in microns). Total distended body length 840. Head length (width) 60 (110); eye length (width) 50 (32). Pronotum length (width) 76 (130). Length of antenna 173; length (width) of antennal segments I 15 (19), II 22 (22), III 30 (12), IV 30 (11), V 26 (12), VI 31 (11), VII 9 (4) and VIII 10 (3). Fore wing length 1090.

#### Etymology.

The species name is an arbitrary combination of two Latin adjective, “*ater*” meaning black, and “*maculosus*” meaning spotted or markings, in reference to the abdominal tergites with many dark brown markings.

#### Distribution.

China (Hunan, Fujian).

#### Remarks.

This species can be distinguished from other members of the genus *Asprothrips* by the variable number and size of brown markings on the abdominal tergites (Figs 2–5). These dark brown markings are obviously not subintegumental pigment because they are present on specimens before and after treatment with NaOH. Intraspecific variation in colour and structure within and between populations is common in Thysanoptera (Mound 2005b). Historically, it was not unusual for thrips taxonomists to describe one species under many different names because of failure to recognise such phenotypic plasticity (Mound 2005a). For example, *Ecacanthothrips tibialis* (Ashmead) had been given 18 different names (Palmer and Mound 1978). Similarly, *Frankliniella occidentalis* (Pergande), the Western flower thrips, has been reported to exist in three colour morphs, with light, dark, and bicoloured forms from different populations or seasons, resulting in at least 16 described species being placed as synonyms of *F. occidentalis* (Mound and Marullo 1996). Environmental conditions are probably of importance in determining such colour differences (Mound 2005a, b). However, *A. atermaculosus* is unusual because the brown markings on III–V and VII–VIII of females vary in number and size within the same population. In this study, we collected mainly at Chaling (Hunan province) and Sanming (Fujian province) respectively, these two localities are approximately 400 km apart. In “Chaling” population, there are eight colour patterns of brown markings on tergites III–V in female (Fig. 5), whereas in the “Sanming” population there are 12 kinds of brown markings (Fig. 4). Despite this, the colour morph with paired markings on tergites I–VIII is dominant and found in both populations. Furthermore, the paired brown markings are stable in their presence on tergites I–II and VI in the sexes. Therefore, much is yet to be learnt about the biological significance of the variation in colour markings of this new species. Such variation also occurs in the female of *A. bimaculatus* Michel & Ryckewaert, which has a pair of dark brown markings on abdominal tergite VI (Michel and Ryckewaert 2014a, b), but in Chinese specimens, these markings are faded and only faintly visible (Tong et al. 2016).

### Asprothrips fuscipennis

Taxon classificationAnimaliaORDOFAMILIA

Kudô

Figs 17–24

Asprothrips fuscipennis Kudô, 1984: 487

#### Material examined.

**CHINA**, Jiangxi province, Jiujiang City, Mt. Lushan (29°33'41"N, 115°58'19"E), 12 females collected from leaves of *Ilex crenata* (Aquifoliaceae), 9.xi.2015, leg. Xiaoli Tong.

#### Diagnosis.

**Figures 17–24.**
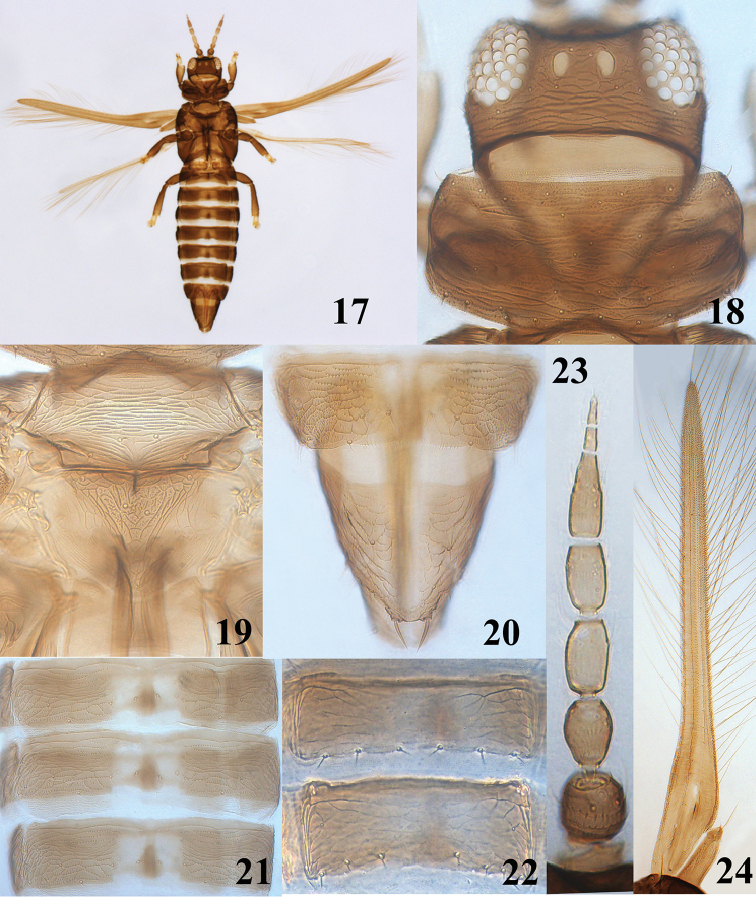
*Asprothrips fuscipennis* Kudô, 1984, female. **17** female adult **18** head & pronotum **19** meso- and metanotum **20** abdominal tergites VIII–X **21** abdominal tergites IV–VI **22** abdominal sternites VI–VII **23** antenna **24** fore wing.

Female full winged and body brown except for all tarsi yellow (Fig. 17). Antennae 8-segmented (Fig. 23); segment II with a subbasal dorsal seta, segments III and IV paler than other segments and each with a forked sense cone, VI longer than IV. Pronotum, meso-and metanotum reticulate and without long setae (Figs 18, 19); tarsi all bi-segmented; fore wing without uniform covering of microtrichia (Fig. 24), first vein with 5–6 proximal and two distal setae, second vein with 7–9 setae; main posterior fringe hairs largely straight. Abdominal tergites I–VII smooth medially and reticulate laterally (Fig. 21); tergite VIII with posterior marginal comb of microtrichia, posterior margin of IX with a pair of long and fine setae medially (Fig. 20) and X with complete longitudinal dorsal split; abdominal sternites III–VII each with three pairs of setae at posterior margin (Fig. 22).

#### Distribution.

Japan and China (Jiangxi).

#### Remarks.

*Asprothrips fuscipennis* Kudô is newly recorded from China in this study. Although Zhang and Tong (1988) reported this species from China, the specimens on which that record was based were subsequently described by Tong et al. (2016) as a new species, *A. bucerus*. These two species are very similar in colouration and structure, but *fuscipennis* can be distinguished from *bucerus* by (1) antennal segment II with a sub-basal dorsal seta (this seta absent in *bucerus*); (2) antennal segment IV shorter than VI (segment IV longer than VI in *bucerus*); (3) posterior margin of tergite IX with a pair of long and fine setae medially (IX with a pair of short horn-like setae directed medially in *bucerus*), and (4) abdominal tergite X with complete longitudinal dorsal split (longitudinal dorsal split incomplete, divided only in distal half in *bucerus*).

## Supplementary Material

XML Treatment for Asprothrips atermaculosus

XML Treatment for Asprothrips fuscipennis
